# Development of a simulation technical competence curriculum for medical simulation fellows

**DOI:** 10.1186/s41077-022-00221-4

**Published:** 2022-08-09

**Authors:** Rami A. Ahmed, Dylan Cooper, Chassity L. Mays, Chris M. Weidman, Julie A. Poore, Anna M. Bona, Lauren E. Falvo, Malia J. Moore, Sally A. Mitchell, Tanna J. Boyer, S. Scott Atkinson, Johnny F. Cartwright

**Affiliations:** 1 Division of Simulation, Department of Emergency Medicine, Indiana University School of Medicine, Indianapolis, USA; 2 Indiana University School of Nursing, Indianapolis, USA; 3 Indiana University School of Medicine, Indianapolis, USA; 4 Department of Anesthesiology, Indiana University School of Medicine, Indianapolis, USA; 5 Level 3 Healthcare, Provo, Utah USA

**Keywords:** Simulation fellow, Simulation technical competence, CHSOS, Sim tech, Simulation technology specialist, Summative assessment, Curriculum

## Abstract

**Background and needs:**

Medical educators with simulation fellowship training have a unique skill set. Simulation fellowship graduates have the ability to handle basic and common troubleshooting issues with simulation software, hardware, and equipment setup. Outside of formal training programs such as this, simulation skills are inconsistently taught and organically learned. This is important to address because there are high expectations of medical educators who complete simulation fellowships. To fill the gap, we offer one way of teaching and assessing simulation technical skills within a fellowship curriculum and reflect on lessons learned throughout the process. This report describes the instructional designs, implementation, and program evaluation of an educational intervention: a simulation technology curriculum for simulation fellows.

**Curriculum design:**

The current iteration of the simulation technical skill curriculum was introduced in 2018 and took approximately 8 months to develop under the guidance of expert simulation technology specialists, simulation fellowship-trained faculty, and simulation center administrators. Kern’s six steps to curriculum development was used as the guiding conceptual framework. The curriculum was categorized into four domains, which emerged from the outcome of a qualitative needs assessment. Instructional sessions occurred on 5 days spanning a 2-week block. The final session concluded with summative testing.

**Program evaluation:**

Fellows were administered summative objective structured exams at three stations. The performance was rated by instructors using station-specific checklists. Scores approached 100% accuracy/completion for all stations.

**Conclusions:**

The development of an evidence-based educational intervention, a simulation technical skill curriculum, was highly regarded by participants and demonstrated effective training of the simulation fellows. This curriculum serves as a template for other simulationists to implement formal training in simulation technical skills.

**Supplementary Information:**

The online version contains supplementary material available at 10.1186/s41077-022-00221-4.

## Main text

Medical simulation fellowship trained faculty have a unique skill set from other medical educators [1–3]. As a result of their expert training in debriefing, simulation curriculum development, educational theory, simulation technical skills, and administration, they are frequently recruited and tasked with leading simulation programs and simulation centers [4]. In an effort to deliver consistent education and meet new accreditation standards, there is a need to standardize simulation fellowship training [5, 6]. Currently, few simulation fellowships include simulation technical skill training and assessment as a formal part of the curriculum and instead focus heavily on the development of expertise in debriefing, case design, and curriculum development [6]. At present these, technical skills are inconsistently taught and organically learned.

Simulation fellowship graduates (hereby called fellowship graduates) acquire the ability to handle basic and common troubleshooting issues with simulation software, hardware, and equipment setup. This is only one skill set that distinguishes fellowship graduates from clinical faculty who utilize simulation with no formal training [7, 8]. This training quickly puts fellowship graduates at the same ability of those simulation pioneers who acquired their skills through experiential, on-the-job learning, and often by trial and error before formal training was available. This combination of skills, frequently called “sim tech skills,” are typically mastered and executed in simulation centers by simulation technology specialists [9]. Simulation technology specialists, or “sim techs,” provide expertise in the setup, execution, and troubleshooting of high-quality simulation training events, which permits the faculty to focus on case-flow, learner assessment, and debriefing [10]. Fellowship graduates are expected to have an advanced skill set to concordantly manage the education, run simulators, and address common equipment issues [5–7, 11].

Fellows typically learn these technical skills in an apprenticeship-style approach through informal training and guidance by experienced simulation technology specialists. Importantly, many established centers with fellowships are staffed with certified simulation experts—CHSOS® (Certified Healthcare Simulation Operations Specialist) and CHSE® (Certified Healthcare Simulation Educator)—who are qualified to teach this skill set [12–16].

Our simulation fellowship curriculum includes formal simulation technical skills training with summative assessments. There is longitudinal exposure to key concepts and training in common simulation technical skills expected of an entry level simulation technology specialist. The educational goal of this training is to provide fellows with a basic skillset to troubleshoot and repair common simulation equipment malfunctions and failures, so as to function independently without sim techs.

### Rationale

We developed this curriculum as a mandatory, formal component of our simulation fellowship. In this report, we describe the design, development, and implementation of the simulation technical skills curriculum between 2018 and 2021, learner assessment and outcomes, and lessons learned regarding iterative modifications. We anticipate that others may adopt and adapt this curriculum component for their specific program needs.

### Educational setting

The program was implemented in 2018 and administered over 3 years. A total of eight simulation fellows joined after completing residency or fellowships in either emergency medicine, pediatric emergency medicine, or pediatric critical care. The Simulation Center at Fairbanks Hall is a collaborative partnership among the Indiana University (IU) School of Medicine, IU School of Nursing, and IU Health System. The Sim Center is over 30,000 square feet (to include inpatient, acute care, and clinic settings) and delivers over 1200 events and 50,000 learner hours of education annually. The Sim Center has 22 full- and part-time staff, including 10 simulation staff with CHSOS® or CHSE® certification, and three with advanced certification (CHSE-A or CHSOS-A) (CHSE-A Certified Health Simulation Educator-Advanced; Certified Health Simulation Operations Specialist-Advanced). The IU School of Medicine is the largest allopathic medical school in the USA, the IU School of Nursing is the largest nursing school in the state, and IU Health System is the largest employer in the state.

### Curriculum design

The initial simulation technical skill curriculum was introduced in 2018 and took approximately 8 months to design and develop by a committee of experts:

- Two simulation faculty members (emergency medicine)
- Three certified simulation operation specialists (2 CHSOS-A; 1 CHSOS)
- One nurse administrator/educator (doctorate in nursing practice, DNP and CHSE-A)
- One simulation educator/administrator (anesthesiology, doctorate in education, EdD)
- One simulation faculty member/administrator (anesthesiology, physician).

Our current fellowship program director previously (2013) executed a comparable basic sim tech-ops program for a simulation fellowship program at a prior institution with a total of 10 fellows from three specialties: emergency medicine, pediatric emergency medicine, and obstetrics and gynecology.

The fellowship program director assembled the committee of simulation faculty and staff and reviewed sources of information regarding simulation technical skill training: the CHSOS content domains and examination blueprint, several simulation tech textbooks, the very limited number of peer-reviewed journal articles on technical skills and training, and written feedback from previous fellows. These resources were evaluated for common overlapping themes. This was further scrutinized through the lens of what would be most practical and beneficial to fellows within the capability of our simulation center.

Kern’s six steps to curriculum development was used as the guiding conceptual framework:

### Kern’s six steps [17]

#### Step 1: problem identification

Current simulation fellows and recent graduates serving as program faculty lack basic technical skills to operate simulation equipment (i.e., hardware and software), troubleshooting when issues occur (i.e., identification and anticipation of problems), repair/fixing the issues (i.e., problem solving), and finding helpful resources (i.e., website, user manuals, how-to videos). Thus, fellows and faculty were dependent on the presence of simulation technology specialists to ensure a successful learning experience. Fellows/faculty frequently contacted simulation technology specialists to consult on technical problems (i.e., disruption to learners) via email, text messaging, and phone calls who were assigned to other simulation events or work duties (i.e., disruption to others, interrupted sim tech attention), and while off work (i.e., disruption of personal time, violation of non-exempt worker employment statutes). The simulation techs voiced to leadership their concerns and frustrations that fellows/faculty required consultation for routine operation of equipment, commonly encountered technical issues, and high-frequency workflow problems, which appeared to be basic skills that could be easily taught and learned. The leadership team acknowledged a curricular gap may exist, which could be filled via an educational intervention.

#### Step 2: needs assessment

Simulation fellows need structured, organized, technical skills training, akin to those basic skills held by simulation technology specialists, to function independently and correct common equipment issues when running simulation sessions for learners. Focused discussions were had by the authors with fellow graduates and simulation technology specialists to identify (a) the desired skills for simulation faculty and (b) the basic skills that technology specialists think simulation faculty should possess. From this qualitative investigation, the outcome of four major themes/categories emerged as knowledge gaps:

1. Hardware/software content and mannequins
2. Technical skills and troubleshooting content
3. Learning management system
4. Task trainer setup, proper care, and handling.

#### Step 3: goals and objectives

This is to enhance the competency of simulation fellowship graduates by including a technical skill curriculum.

#### Step 4: educational strategies

Educational strategies should be applied in person immersive training (the intervention of experiential learning on the hardware, software, and mannequins), asynchronous reading (pre-intervention of user manuals, “how to” guides, manufacturer websites), longitudinal skill utilization (microlearning throughout the year-long fellowship as embedded activities during highly technical simulation sessions), summative competency testing (post-intervention of the immersive training).

#### Step 5: implementation

The curriculum was designed and implemented using a scaffolding framework such that foundational information was taught first and repeated when new, related topics were presented. For example, how to perform a pre-use check on the mannequins preceded troubleshooting common problems that occur during use. Also, content was grouped to address high fidelity mannequins separate from low-fidelity task trainers. Asynchronous pre-reading prepared fellows for the experiential learning sessions. Summative testing allowed fellows to demonstrate achievement, receive feedback from faculty, correct misunderstandings/errors, and reflect on the curriculum. Additionally, the overall design served as a hidden curriculum since fellows were training to become sim educators while serving as learners in a sim session complete with pre-work, scenarios, and summative testing stations.

Resources/inputs: simulation technology specialist expertise, simulation faculty expertise, leadership support, simulation center resources, simulation tech textbook review, simulation technical training literature review, educational courses, and certification review. Deliverables/outputs: lesson plans, time allotment/scheduling, resources list, and grading checklists. Iterative improvement based on faculty and fellow feedback.

#### Step 6: evaluation

Programmatic evaluation of outcomes are as follows: internally developed checklists, summative testing stations, faculty feedback, and fellow feedback. High scores on the summative stations and positive feedback from faculty and fellows support that the educational intervention was successful and that the learning goal was achieved to enhance the competency of simulation fellowship graduates by including a technical skills curriculum.

As a result of the above analysis, the developed curriculum was organized into 4 main themes. These themes are further explained and illuminated below using a combination of text, tables, and figures.

**Fig. 1:**
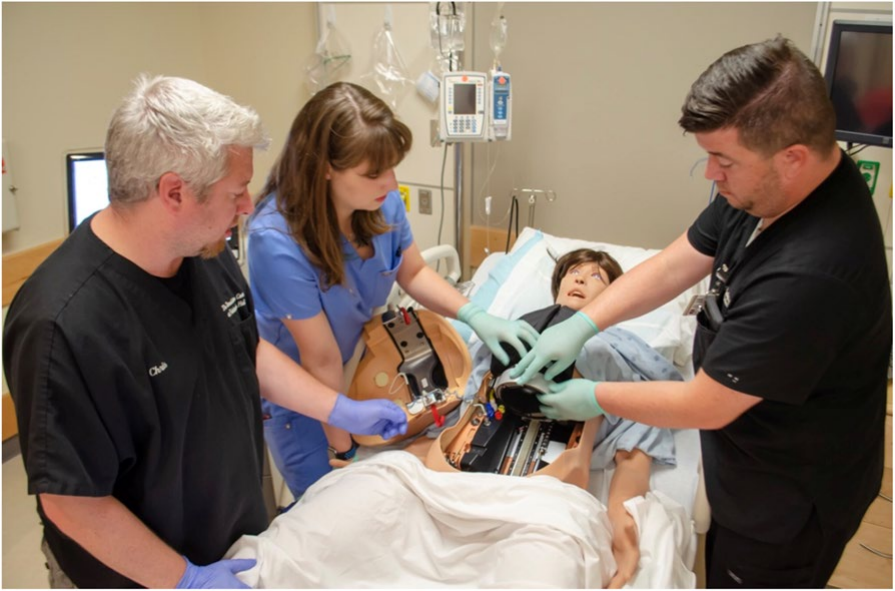
Medical simulation fellow participating in immersive formative training on high-fidelity simulator connectivity and troubleshooting common issues

**Fig. 2:**
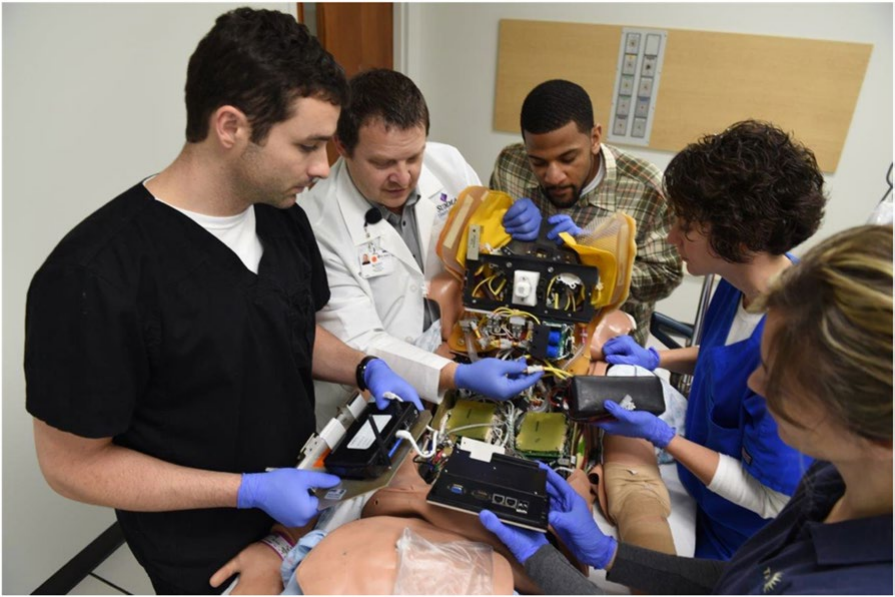
Simulation technology specialist providing formative training to simulation fellows and staff on hardware, technical skills, and troubleshooting common issues

1. ***Hardware/software content and mannequins***
  The content related to mannequin-based skills was designed as a broad exposure to various mannequins to provide an optimal foundation for the fellows. The training included an overview of high-fidelity mannequins, including adult, adolescent, newborn, and birthing mannequins The simulation center primarily uses Laerdal® high-fidelity mannequins, except for birthing mannequins which are from CAE® (Canadian Aviation Electronics) and Gaumard®. Almost exclusively using simulators from one manufacturer allows the center to run one software program across all computer control stations and simulated patient monitors. This facilitates quicker onboarding for simulation technology specialists and faculty and for these operators to have a consistent experience when running sessions. Also, a single-software system allows for easier and less time-consuming tasks of updating and networking.
  Introductory content for the mannequins followed the content outline in Domain II (sections A & B) of the CHSOS® examination blueprint [13]. This included basic software and user interface training, including turning on the simulator’s software system, manipulation of vital signs, physical exam findings, utilization of audio-representing the mannequin’s voice, and display of medical images (i.e., ECGs (electrocardiogram), X-rays, CT (computed tomography) scans) on simulated patient monitors. After the fellows were introduced to these topics and provided time to perform all functions, they were given a demonstration on the external hardware/parts of the high-fidelity mannequins for how to do a pre-use check: head-to-toe assessment; inspection of the skin for damage, moisture, or left over moulage; ECG and defibrillation posts; and loose or exposed wiring. The next topic focused on the various internal parts that need to be inspected pre-use: batteries, defibrillation wiring, ECG wiring, lung bladders, fluid reservoirs, pneumatic connections, and limb connections. The inspection process culminated in a demonstration on how to replace/repair the items mentioned above. The fellows were then each given the opportunity to perform repairs or replacement (see Figs. 1 and 2). A question-and-answer session followed to assess knowledge retention, provide clarification, and revisit mannequin inspection steps. This provided a transition to the next section of the curriculum.
2. ***Technical skills and troubleshooting content***
  This portion of the curriculum provides an opportunity to review and demonstrate the previous foundational lessons while building a deeper understanding of common troubleshooting situations. In many simulation centers, simulation educators do not have the support for hiring a simulation technology specialist. Therefore, it is crucial that sim educators possess certain technical skills and perform troubleshooting techniques at a basic level as written in user manuals from the original equipment manufacturer (OEM). Fellows and simulation technology specialists review the OEM user manuals. Subsequently, a few additional steps that experienced simulation technology specialists complete are reviewed to promote efficiency and success when fellows may be working autonomously or with limited tech support.
  Based on principles in Domain II (section C) of the CHSOS® examination blueprint, the fellows were shown how to work through troubleshooting and then practiced these skills. This portion of the curriculum started with common network connectivity issues. While this portion is specific to our sim center Internet options and mannequins, we recognize and emphasize to the fellows that alternative technologies (i.e., hardwire computer to mannequins with ethernet) may be used and that mannequin-specific connectivity differs among and within manufacturers. Also, we recognize that highly technical terminology and hardware components may differ among countries and suggest referring to the country of use in the OEM manuals.
  The mannequins at our center rely on a WLAN (wireless local area network) or wired LAN (local area network). Some mannequins (i.e., pediatric, baby, and neonate) rely on a CAN BUS (controller area network omnibus) computing system. We use an internal network, or intranet, to ensure mannequins and control computers are linked only to each other. Having functional knowledge of these networking differences and how the mannequins and computers connect are key responsibilities of simulation technology specialists. A knowledge gap in this area leads to a high number of mistakes and ultimately a high incidence of failure when using these mannequins. Mistakes can often be avoided by following the OEM user manual. The fellows were shown how to connect to an internal network and how to configure a mannequin to that respective network. Once shown, they practiced multiple times. Next, fellows were taught common issues with physical disconnection of the simulators, how to manage this, how to address it mid-simulation, and how to re-establish the connection.
  The high-fidelity birthing mannequins from CAE® and Gaumard® utilize a WLAN connection and an operating system that is web-based (software that runs via an Internet browser). Of note, Internet access is not needed to operate these mannequins. They run via offline functionality built into the software architecture in programs such as Google Chrome® or Mozilla Firefox®. Fellow training with the birthing mannequins included common internal hardware setup and troubleshooting. The session focused heavily on the OEM instructions written in the manuals that were demonstrated during hands on practice.
  While a hardwired system is most reliable and stable, we also covered wireless connections to address mannequins that only connect wirelessly. Additionally, the benefit of mobility was emphasized. For instance, in situ events where the mannequins are blocked from joining the hospital network via ethernet and WIFI (wireless fidelity), which requires knowledge of a stand-alone network.
3. ***Learning management system***
  The learning management system training was designed to provide the fellow directives on how to sign into the system, record playback, and collect data needed for learner performance assessment and simulation-based research. Fellows were previously introduced to video playback during debriefing both as learners and instructors. Here, we covered the control room aspects to ensure that events are recorded and available for playback, assessment, and research. Again, we used the OEM as an instructional guide and practiced the steps repeatedly with other fellows/faculty/staff serving as learners in a mock simulation scenario.
  Although many sim centers may lack this robust educational technology, it is important to recognize the potential impact on teaching and learning. Learner debriefing is enriched by incorporating video playback, which aids in accurate depiction of the scenario events and drives discussion [18]. Also, learner self-reflection as an instructional method is enhanced by having learners watch and annotate their own recordings. To emphasize this point, fellows took turns acting as the learner and the instructor in sequential scenarios, reviewed their learner performance playback, and reviewed their instructor debriefing playback. A group discussion and debrief of the debrief included fellow self-reflection, faculty observations, and sim tech feedback. We then discussed alternative, low-budget, portable recording devices (i.e., tablets, smart phones, laptop with external camera, external microphones) that may be used in lieu of an installed, permanent event capture system, and still meet minimum requirements for recording and playback. The self-reported knowledge base and confidence level of the fellows on use of these devices was high, and thus, we did not practice these skills.
4. ***Task trainer setup, proper care, and handling***
  The task trainer portion of the curriculum was developed to ensure the fellows were able to demonstrate proficiency in setting up, maintaining, cleaning, breaking down, and troubleshooting the most common issues of frequently used medical task trainers (see Table 1). This session included a review of task trainer user manuals, an overview of the internal mechanics/parts, setup instructions, how to keep task trainers free of mold and leaks, how to drain fluids, and proper storage. Once these basics were addressed, common troubleshooting issues were discussed for each step. Fellows then practiced with the task trainers. This hands-on practice is essential for discovering “what is under the skin” of the task trainers since sim educators may be uncomfortable disassembling a costly piece of equipment and unfamiliar with setup, maintenance, and storage.

**Table 1:** Task trainers reviewed

Trainer	Setup	Teardown
**Central line trainer**	**Inspection of insert and priming of fluids for insert**	**Inspection of insert and priming of fluids for insert. Clean and disinfect all surfaces per OEM manual. Placed in appropriate storage case and stowed in appropriate position (per OEM manual)**
**Arterial line trainer**	**Filling fluid reservoir, installing new artery tubing, inspecting, and installing arm tissue, priming fluid system**	**Draining of fluid reservoir, remove arm tissue, install new artery, add mixture of sterile water and 1–2cc isopropyl alcohol, prime system, repeat until system plumbing is clear, drain system. Clean and disinfect all surfaces per OEM manual. Place trainer in appropriate case for storage**
**Lumbar/epidural trainer**	**Inspection of tissue insert, priming, and or removal of air depending on LP or EP usage**	**Inspection of insert, prime fluid, or remove air. Place in appropriate storage case and stowed in appropriate position (per OEM manual)**
**Airway/intubation heads**	**Inspection of overall appearance, tears in tongue, broken teeth, holes/tears in lung bladders, lubricate airway with OEM-approved airway lubricant**	**Inspection of overall appearance, tears in tongue, broken teeth, holes/tears in lung bladders, wipe away excess airway lubricant, Clean and disinfect all surfaces per OEM manual. Place in appropriate storage case**
**Trauma man**	**Inspection of simulator skin, selection and installation of appropriate tissue insert**	**Inspection of simulator skin, removal of used tissue inserts. Clean and disinfect all surfaces per OEM manual. Place in appropriate storage case**

*OEM* original equipment manufacturer

### Implementation and assessment of learning

**Fig. 3:**
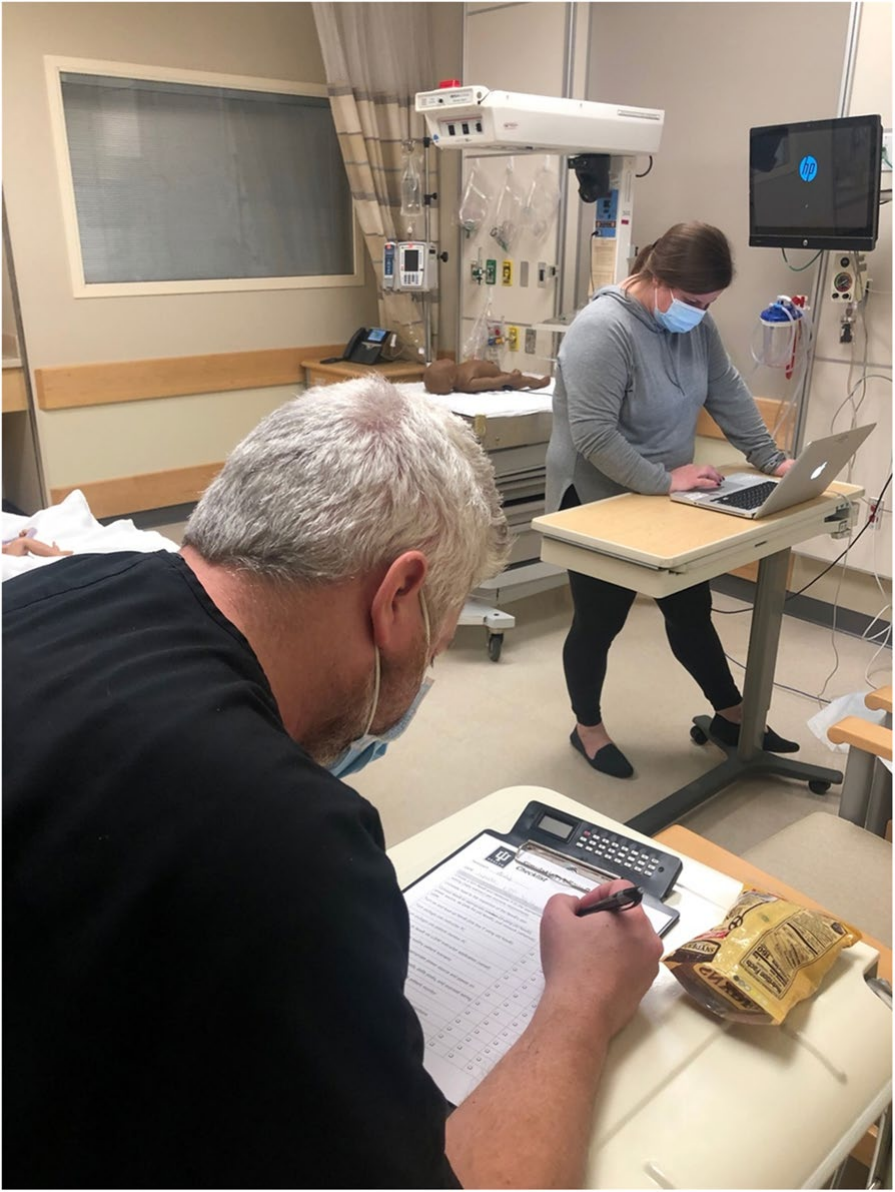
Simulation fellow undergoing summative testing on simulator software and troubleshooting

**Fig. 4:**
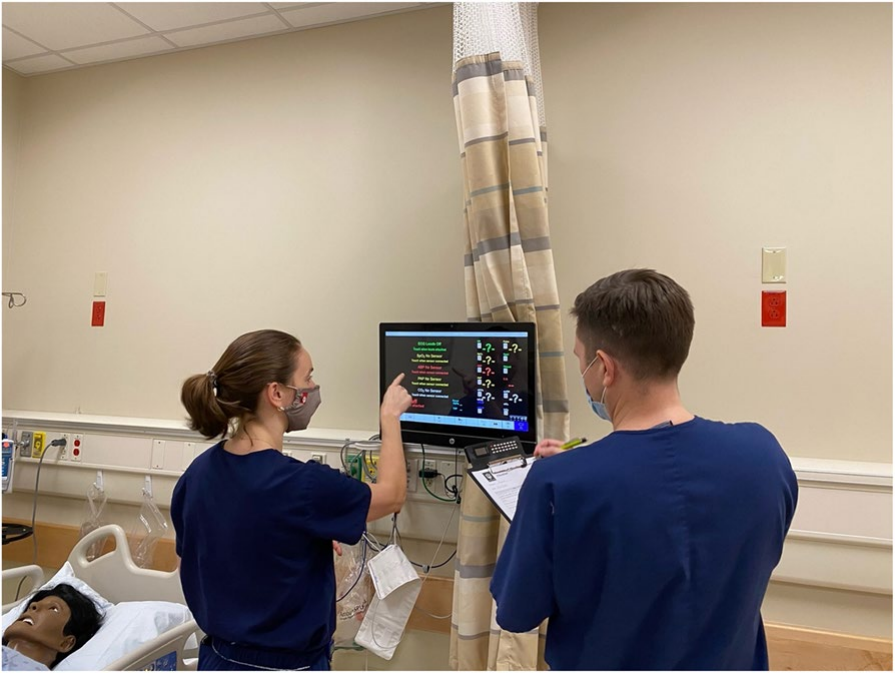
Simulation fellow undergoing summative testing on simulator software and troubleshooting

**Fig. 5:**
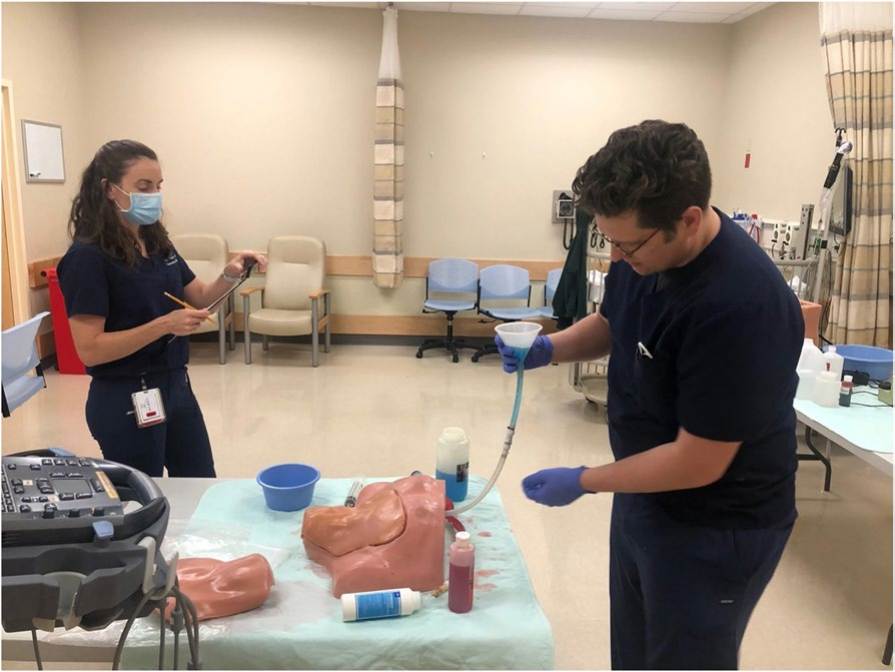
Simulation fellow undergoing summative testing on task trainer setup and maintenance

This program consisted of 4 days of 3-h formative sessions and a fifth day for 3 h of summative testing (12 h formative, 3 h summative, 15 h total) spread over a 2-week period (see Table 2). The fourth 3-h session was a review period where fellows engaged in discussion, asked questions, and practiced technical skills. The course concluded with a 3-h testing session that covered three stations. Each station was staffed with 1 to 2 faculty or simulation technology specialists to assess the fellows’ performance (see Figs. 3, 4, and 5). No formal rater training was incorporated into this curriculum.

**Table 2:** Outline of simulation technical curriculum for fellows

	Description	Outcome
Day 1:		
OBJECTIVES	° Discuss simulator selection process depending on simulation scenario objectives/needs ° Setup and prepare Laerdal® simulator hardware & software ° Setup & shutdown of router-based mannequins ° Setup & shutdown of link box-based mannequins ° Navigate and apply knowledge in order to troubleshoot simulator connectivity issues ° Review and discuss features specific to each high-fidelity patient simulator ° Discuss and demonstrate proper care and handling of each high-fidelity patient simulator	
INFORMATION	Laerdal® LLEAP guide/manual Laerdal® SimMan 3G manual Laerdal® SimBaby manual Laerdal® SimNewB manual	Student will be given specific pages of each manual in order to reference steps taught during this lesson
ACTIVITY	Break into equal sized groups and have each student run through the following: ° Simulator selection and setup procedures ° Simulator start up and shut down procedures ° Simulator inspection and prep for usage ° Troubleshooting of hardware, software, networking ° Care and handling before and after simulator usage	Each student will have the opportunity to practice and complete a full setup and shutdown of each Laerdal® high-fidelity simulator
DAY 2		
OBJECTIVES	° Operation of Laerdal® LLEAP software ° Basic scenario building/programming within LLEAP ° Setup and operation of birthing simulator ° Operation of CAE® Muse software	
INFORMATION	Laerdal® SimMan 3G manual Laerdal® SimBaby manual Laerdal® SimNewB manual Laerdal® SimJr manual CAE® Lucina manual	Student will be given user guides for each simulator in order to reference steps taught during this lesson
ACTIVITY	Break into equal sized groups and have each student run through the following: ° Navigating and understanding important functions within LLEAP user interface ° Basic programming within LLEAP SimDesigner ° Operation and navigation of the Muse platform	Students will be able to operate and navigate the LLEAP and CAE® software to successfully run a Laerdal® or CAE® simulator for a sim scenario
DAY 3		
OBJECTIVES	° Operation of B-Line® SimCapture Learning Management System ° Setup, operation, maintenance, and troubleshooting of various task trainers	Student will be able to demonstrate the ability to successfully setup the SimCapture recording system in order to record a sim session Students will be able to choose, setup, troubleshoot, and tear down various task trainers
INFORMATION	Simulab® A-Line Arm manual Simulab® LP/EP manual Simulab® central line man manual Simulab® TraumaMan manual CAE® Blue Phantom CVL manual Laerdal® & 3B Scientific ®airway trainer manual	Student will be given user guides for each simulator in order to reference steps taught during this lesson
ACTIVITY	Break into equal sized groups and have each student run through the following: ° Navigating and understanding important functions within B-Line’s® SimCapture software ° Selection, setup, troubleshooting, maintenance, and clean up for each of the task trainers	Each student will be able to demonstrate proficiency in setting up, maintaining, cleaning, breaking down, and troubleshooting the most common issues from commonly used medical task trainers
DAY 4		
OBJECTIVES	° Review each of the previous day’s activities	Students can go over any of the course’s activities they feel they need more practice on
DAY 5		
OBJECTIVES	° Exam day ° Test station 1 ° Test station 2 ° Test station 3

Checklists were used to rate the fellows in real time. The checklists were developed from established best practices in a variety of simulation skill areas, the user manuals for the products, and expert guidance from simulation technology specialists (see Additional file 1). A score of 80% was predetermined to be the minimum passing score.

### Program evaluation

#### Assessment of learning results

Fellows were tested on three of the four summative checklists. All fellows scored nearly 100% on all stations, and none scored below the benchmark of 80%.

#### Course feedback summary

Fellows provided open response feedback regarding the strengths and weakness of the curriculum. The first iteration of the course was executed over 8 weeks. The fellow’s major feedback that there was too much time between training sessions. The course was compressed so that the formal training sessions were all completed within 1 week. The final review and test session were held the following week. We received positive feedback on the compressed schedule the following year. Overwhelmingly positive feedback was received for the final review session as it ensured their ability to demonstrate skills, review troubleshooting approaches, and answer final questions.

Overall, the fellows reported appreciating the immersive environment that focused heavily on hands on skills (deliberate practice with expert coaching) with minimal lecture/didactics.

## Discussion

The authors report the development, implementation, lessons learned, and feedback on an innovative curriculum for medical simulation fellows focused on technical skill development (aka “sim tech skills”). The immersive approach to the utilization of a variety of simulators, task trainers, and troubleshooting common issues with software and hardware delivered in a deliberate practice/mastery learning instructional method [19] over several sessions proved to be a successful approach with our fellows. This success was partially a result of using experts/coaches providing immediate, real-time feedback with sessions that were executed in a timeframe that facilitated progressive learning within the fellow’s zone of proximal development [20]. This immersive approach, in combination with reducing the time gap between formative training sessions and summative testing sessions, likely explains the high scores demonstrated by the fellows in our program.

Many fellowship programs preach deliberate practice and mastery learning to their learners that come through their labs yet continue to train their fellows in an apprenticeship model where the fellows inconsistently participate, get haphazard formative feedback, are infrequently formally trained, and rarely administered summative tests [6, 7]. As a result, the simulation literature regarding curriculum development and research in this particular area of simulation is very limited. Overall, fellows strongly valued the time spent getting hands-on experience, receiving real-time feedback, and working through specific tasks and stations. Curricula like this could lead to further standardization of training for simulation fellows globally and potentially prepare fellows to take the CHSOS® exam. Furthermore, after completion of the course, the fellows intermittently worked in the role of sim tech to setup, run scenarios, and breakdown skill stations for various groups of learners. This was done intentionally to minimize skill decay [21, 22] and ensure maintenance of training prior to fellowship graduation. We continually emphasized the utility of OEM manuals and that the process of learning would be transferable to new environments, equipment, and systems.

### Lessons learned and recommendations

The scheduling of this curriculum has been through several iterations. In its first year, fellows were asked to attend a total of four half-days of training, spread over 2 months, with a cumulative test in month 9 of a 12-month fellowship. Based on fellow feedback, this large time gap between sessions resulted in significant skill decay, anxiety, and reiteration of previous lessons before getting to the goals and objectives of the scheduled training for the day. As a result, the course was then altered to be a 1-week intensive curriculum with four half-days of education culminating in a comprehensive exam on a fifth day the following week. Additionally, the fellows provided the feedback that they wanted some overlap or review from the previous day of training as a part of the lesson plan for each day of the curriculum, concluding with a formative review day that provided an opportunity to revisit the entire curriculum.

In the second year, we included non-fellows in the training session, such as ancillary (non-sim tech) simulation staff, faculty/staff from other departments who infrequently utilized simulation, and entry level simulation technology specialists. Fellows reported that this took away from their experience and gave them less personalized attention. They suggested a homogenous cohort to ensure the training focused on their needs as future faculty members and simulation educators. We subsequently changed this curriculum to only train simulation fellows, which garnered positive feedback and an increased comfort level with the material before proceeding to the summative testing period.

Our institution may be unique in its ability to employ multiple simulation faculty and certified simulation staff to host this curriculum. We were able to educate and assess three fellows in the first year and four fellows in the second year of this program. However, not all institutions will have the expertise or resources to implement this simulation technical competence curriculum. We recommend advanced planning to ensure adequate support. Additionally, we recommend summative assessment stations be run in parallel when testing multiple learners for quick turn-around and set-up. An alternative is to run stations sequentially where all examinees complete Station 1 and then move on to Station 2. Smaller institutions may utilize the expertise and availability of mannequin sale representatives to provide exposure to equipment not available at the institution.

### Next steps

The faculty continue to refine the curriculum based off current fellow and graduate feedback on the content and delivery of this training. Future research could assess the impact of this curriculum on fellow confidence and performance during simulation education events and post-fellowship, in their leadership roles. Long-term impact may be measured by graduate scholarship published works, grants awarded, simulation center certification attainment, personnel certification attainment, new centers designed, and opened.

## Conclusion

The development of a novel simulation technical skills curriculum was highly regarded and effective for the training of simulation fellows. This curriculum provides a template for other fellowships to provide formal training in simulation technical skills to future simulation faculty leaders.

## Supplementary Information

**Additional file 1.** Sim tech skills fellow exam.

## Data Availability

The datasets used and/or analyzed during the current study are available from the corresponding author on reasonable request.

## Abbreviations

CHSOS®: Certified Healthcare Simulation Operations Specialist
CHSE®: Certified Healthcare Simulation Educator
IU: Indiana University
CHSE-A: Certified Healthcare Simulation Educator-Advanced
CHSOS-A: Certified Healthcare Simulation Operations Specialist-Advanced
CAE: Canadian Aviation Electronics
ECGs: Electrocardiograms
CT: Computed tomography
OEM: The original equipment manufacturer
WLAN: Wireless local area network
LAN: Local area network
CAN BUS: Controller Area Network Omnibus
WIFI: Wireless fidelity
