# Supplementary material for: Development of a simulation technical competence curriculum for medical simulation fellows
Source: Adv Simul (Lond). 2022 Aug 9;7:24. doi: 10.1186/s41077-022-00221-4 (PMC9361680; doi:10.1186/s41077-022-00221-4)
Supplement: Supplementary file 1 — Additional file 1. Sim tech skills fellow exam. [file 41077_2022_221_MOESM1_ESM.docx]

Participant: _____________________

DATE: _____________

| Task | Pass | Fail | N/A |
| --- | --- | --- | --- |
| Connect SimMan 3G to appropriate power source, air (only needed if using older 3G’s that have bad compressors) | ❑ | ❑ | ❑ |
| Inspect SimMan3G for loose leads, defib posts, and exposed wiring | ❑ | ❑ | ❑ |
| Turn on and boot up SimMan 3G | ❑ | ❑ | ❑ |
| Turn on/login into instructor PC | ❑ | ❑ | ❑ |
| Turn on/login into patient monitor PC | ❑ | ❑ | ❑ |
| Connect to 3G via LLEAP instructor application (select WiFi) | ❑ | ❑ | ❑ |
| Start a manual healthy patient scenario | ❑ | ❑ | ❑ |
| Notice and diagnose a simulator disconnect | ❑ | ❑ | ❑ |
| Utilize a “technical time out” or embedded participant to assist in connecting an ethernet cable or virtual pt monitor | ❑ | ❑ | ❑ |
| If ethernet cable & virtual pt monitor does not solve the problem, a technical time out is a must and the entire system should be rebooted | ❑ | ❑ | ❑ |
|  | ❑ | ❑ | ❑ |
|  | ❑ | ❑ | ❑ |
|  | ❑ | ❑ | ❑ |
|  | ❑ | ❑ | ❑ |

Participant: _____________________

DATE: _____________

| Task | Pass | Fail | N/A |
| --- | --- | --- | --- |
| Choose a SimJr and maneuver it to the appropriate setting per scenario requirements | ❑ | ❑ | ❑ |
| Connect SimJr to appropriate link box, power source, air (optional), and network (ethernet) cable | ❑ | ❑ | ❑ |
| Inspect SimJr for loose leads, defib posts, and exposed wiring | ❑ | ❑ | ❑ |
| Turn on and boot up SimJr link box | ❑ | ❑ | ❑ |
| Turn on/login into instructor PC | ❑ | ❑ | ❑ |
| Turn on/login into patient monitor PC | ❑ | ❑ | ❑ |
| Connect to SimJr via LLEAP instructor application (wired) | ❑ | ❑ | ❑ |
| Start a manual healthy patient scenario | ❑ | ❑ | ❑ |
| Notice and diagnose a simulator disconnect | ❑ | ❑ | ❑ |
|  | ❑ | ❑ | ❑ |
|  | ❑ | ❑ | ❑ |
|  | ❑ | ❑ | ❑ |
|  | ❑ | ❑ | ❑ |

Participant: _____________________

DATE: _____________

| Task | Pass | Fail | N/A |
| --- | --- | --- | --- |
| Choose a Sim NewB and maneuver it to the appropriate setting (baby warmer) per scenario requirements | ❑ | ❑ | ❑ |
| Complete head to toe inspection of the NewB’s skin | ❑ | ❑ | ❑ |
| Connect NewB to appropriate link box (if using old NewB), power source, air (only for old NewB), and network (ethernet) cable | ❑ | ❑ | ❑ |
| Turn on and boot up NewB (link box if using old NewB) | ❑ | ❑ | ❑ |
| Turn on/login into instructor PC | ❑ | ❑ | ❑ |
| Turn on/login into patient monitor PC | ❑ | ❑ | ❑ |
| Connect to NewB via LLEAP instructor application (wired) | ❑ | ❑ | ❑ |
| Start a manual healthy patient scenario | ❑ | ❑ | ❑ |
| Connect Lucina to appropriate power source and power on | ❑ | ❑ | ❑ |
| Inspect Lucina for loose leads, defib posts, and exposed wiring | ❑ | ❑ | ❑ |
| Turn on Lucina’s instructor PC | ❑ | ❑ | ❑ |
| Start Muse software and confirm Lucina’s connections | ❑ | ❑ | ❑ |
| Configure Lucina based on scenario template | ❑ | ❑ | ❑ |
| Troubleshoot connectivity &seating of fetus | ❑ | ❑ | ❑ |

Participant: _____________________

DATE: _____________

| Task | Pass | Fail | N/A |
| --- | --- | --- | --- |
| Setup LP/Epidural trainer by inserting appropriate insert | ❑ | ❑ | ❑ |
| Inspect assembled trainer for major leaks and excess air in insert | ❑ | ❑ | ❑ |
| Prime insert with additional fluid and ensure functionality | ❑ | ❑ | ❑ |
| Setup the Simulab A-line trainer appropriately | ❑ | ❑ | ❑ |
| Inspect and Identify A-line insert for leaks | ❑ | ❑ | ❑ |
| Swap out used artery and tissue insert (confirm no leaks are present) and confirm function of task trainer for use | ❑ | ❑ | ❑ |
| Setup Blue Phantom or Simulab CVL trainer on table | ❑ | ❑ | ❑ |
| Inspect (using Ultrasound) for appropriate image | ❑ | ❑ | ❑ |
| Remove excess air in order to prime insert properly | ❑ | ❑ | ❑ |
| Add additional fluid if necessary | ❑ | ❑ | ❑ |
|  | ❑ | ❑ | ❑ |
|  | ❑ | ❑ | ❑ |
|  | ❑ | ❑ | ❑ |
|  | ❑ | ❑ | ❑ |

Participant: _____________________

DATE: _____________

| Task | Pass | Fail | N/A |
| --- | --- | --- | --- |
| Login to B-Line | ❑ | ❑ | ❑ |
| Setup recording for specific scenario & group | ❑ | ❑ | ❑ |
| Check audio and view settings | ❑ | ❑ | ❑ |
| Start recording & make a few annotations | ❑ | ❑ | ❑ |
| Stop recording and close session | ❑ | ❑ | ❑ |
| Login & pull up recorded session in debrief room | ❑ | ❑ | ❑ |
|  | ❑ | ❑ | ❑ |
|  | ❑ | ❑ | ❑ |
|  | ❑ | ❑ | ❑ |
|  | ❑ | ❑ | ❑ |
|  | ❑ | ❑ | ❑ |
